# Sugar Analysis Using Hydrophilic Liquid Chromatography Combined with Raman Spectroscopy

**DOI:** 10.1021/acs.analchem.4c06076

**Published:** 2025-06-14

**Authors:** Yu-Sheng Chen, Hirotsugu Hiramatsu

**Affiliations:** Department of Applied Chemistry and Institute of Molecular Science, 34914 National Yang Ming Chiao Tung University, Hsinchu 30010, Taiwan

## Abstract

The combination of chromatography and Raman spectroscopy is challenging because the eluate flows fast and limits the accumulation time of weak Raman signals. The total reflection of light in a liquid core waveguide (LCW) can enhance the Raman signal intensity and thus solve this problem. We used a vertical flow technique to form an LCW with a sample solution and combined Raman spectroscopy with hydrophilic liquid chromatography (HILIC). Additional techniques were used to eliminate the strong Raman signals attributed to the organic solvent used for HILIC. We report the HILIC-Raman analysis of five sugars (fructose, glucose, sucrose, maltose, and trehalose). These sugars were eluted over 11 min (fructose at 36.5 min and trehalose at 47.4 min) and identified by checking the Raman spectra. The limit of detection (LOD) was 60–80 μg mL^–1^. The HILIC-Raman technique was also applied to analyze the sugar content of honey. The content of fructose, glucose, and sucrose in wt % was determined to be 39.9 ± 0.6, 26.5 ± 1.8, and 0.9 ± 0.5, respectively, and these values agreed with those determined using LC with the refractive index detector. The HILIC-Raman technique offers a novel approach of the quantitative analysis of solutes in the LC eluate.

## Graphical abstract

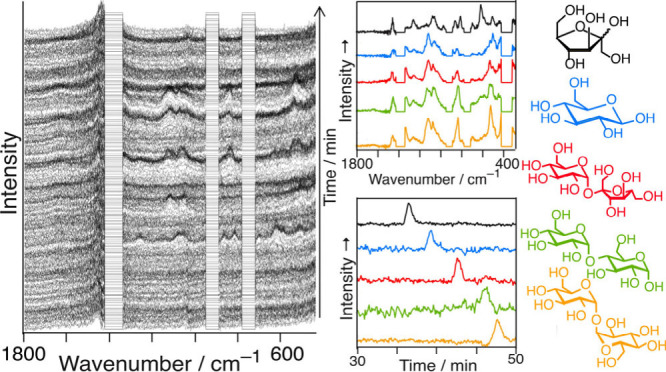

Liquid chromatography (LC) is a popular technique for separating molecules in complex systems by exploiting differences in molecular properties, such as hydrophobicity and hydrophilicity.^1^ Following separation, molecules in the LC eluate are detected and identified. Mass analysis techniques (e.g., LC–mass spectrometry) have been employed extensively for this purpose. Besides, the combination with other spectroscopic techniques has also expanded the utility of LC.^2^ Vibrational spectroscopy is suitable for the identification and characterization of molecules in LC eluates; in particular, infrared (IR) spectroscopy, in which different molecules exhibit distinct spectral patterns because of differences in their molecular structures, has been applied for this purpose.^3^

The combination of Raman spectroscopy, as another vibrational spectroscopy, with LC has been limited because of challenges with detecting the signals of molecules in the LC eluate with a limited data acquisition time. Raman signals of molecules in a flowing LC eluate cannot be readily recorded because of their intrinsic weakness. Established resonance Raman and surface-enhanced Raman scattering (SERS) techniques have been adopted to solve this problem. SERS is suitable for sensitive detection.^4^ These techniques have also been used for the practical detection of the vibrational signals of molecules in LC eluates.^5^ However, these signal enhancement methods require specific conditions and are thus not necessarily universal. Therefore, nonresonance Raman spectroscopy is desired for this type of experiment because it can be applied to any molecule, as no specific conditions, such as an appropriate electronic state(s) or an affinity to metal nanoparticles, are required.

A liquid core waveguide (LCW) enables sensitive detection of optical signals, such as fluorescence.^6^ An LCW confines the excitation beam and signals via total internal reflection, thereby increasing the efficiencies of signal generation and collection. Total internal reflection of light occurs at the interface of two materials, from the material with a larger refractive index (*n*) to that with a smaller *n*. When aqueous samples (*n* = 1.33) are used as the liquid core, fluoropolymers (*n* = 1.29)^7^ and air (*n* = 1.00) can be used as the clad.

A fluoropolymer LCW has been used to combine Raman spectroscopy with LC^8^ and size-exclusion chromatography^9^ by magnifying the Raman signal (up to 1000-fold).^10^ The performance of this technique was recently improved by minimizing interference from bubbles in the fluoropolymer tube by using a 3D-printed degassing module.^11^ Air is also available as a clad to surround a liquid sample. An LCW has been fabricated as a vertical liquid column spouting from a pinhole (i.e., vertical flow (VF) technique).^12,13^ The Raman signal was enhanced up to 168 times when a 60-μm pinhole was used.^14^ The VF technique has also been employed to combine reverse-phase LC with Raman spectroscopy (RPLC-Raman).^15^

RPLC-Raman has been applied to analyze the sugars in honey.^16^ Raman detection is advantageous over other methods, such as refractive index, pulsed amperometric detection, and evaporative light scattering techniques, for sugar analysis^17^ because Raman signals are structure specific. Additionally, derivatization is not necessary to modify the sample hydrophobicity.^18^ A remaining problem associated with RPLC-Raman is interference from the intense Raman bands attributed to organic solvents. Raman spectroscopy is compatible with H_2_O, which has a small Raman cross-section, as the running solvent. RPLC analysis of several sugars (fructose, glucose, sucrose, maltose, and trehalose [abbreviated as Fru, Glu, Suc, Mal, and Tre, respectively]) allowed separation within 2.5 min^16^ with moderate resolving power by using H_2_O as the mobile phase.

Hydrophilic liquid chromatography (HILIC), which is suitable for sugar analysis,^19^ uses a polar column and an aqueous–organic (typically rich in the organic solvent) mobile phase.^20^ The combination of HILIC and Raman spectroscopy is considered useful for analyzing hydrophilic compounds. An expected challenge is strong interference from the Raman bands of organic solvents. In other words, the HILIC-Raman experiment cannot be executed simply by replacing the RPLC column with that for HILIC.

In an experiment combining LC and IR absorption spectroscopy (LC-IR), the eluate is deposited on a substrate as microdroplets using a microdispenser, and the solvent is removed by evaporation.^21^ Microdeposited solute molecules are analyzed via microscopy. This procedure facilitates data sampling of nonvolatile solutes. While this technique can simply and effectively prevent solvent interference, it is not necessarily applicable if solvent removal has a critical effect on molecular structures (for instance, solvent water stabilizes structures and is necessary for protein functions^22^). Additionally, stepwise chromatography and spectroscopy (offline) measurement decreases the throughput of the experiment.

To conduct HILIC and Raman detection successively online, we proposed a novel approach using a spatial line rejection mask to selectively eliminate the Raman bands of organic solvents.^14^ The mask blocks the optical signal without affecting sample flow in the chromatograph and is suitable for combining HILIC with Raman spectroscopy. In this study, we enable the HILIC-Raman experiment for the first time and apply it to the sugar analysis.

## Experimental Section

### HILIC-Raman Spectrometer

Figure 1a shows a schematic diagram of the apparatus.^14^ It consists of a liquid chromatograph (PU-4180-LPG, JASCO) with a high-performance LC (HPLC) column filled with a polyamine-bonded silica gel as the stationary phase (Cosmosil Sugar-D, Nacalai Tesque; 20 mm × 150 mm) for sugar analysis. The LC apparatus includes an ultraviolet–visible (UV–Vis) absorption probe with a single-channel detector (UV-4075, JASCO) and a multichannel photodiode array detector (DAD) (MD-4010, JASCO). The LC eluate is fed into a VF sampling unit for Raman experiments (Figure 1b). The VF unit consists of a fluorinated ethylene propylene sheet with a φ1 mm hole and a φ60 μm pinhole (red), flanking silicone sheets (blue), and a slide slip (black). These layers are stacked and fixed with a screw in a polyetheretherketone (PEEK) holder (brown). The sample liquid flows into the holder, passes through the cavity in the layer structure, and spouts from the 60-μm pinhole (orange). An excitation laser beam focuses on the pinhole via a water-immersion microscope objective (LUMPLFLN40XW, Olympus; NA 0.80). The diameter of the focal point is less than 10 μm (data not shown), allowing the laser beam to enter the sample column and induce Raman scattering. Total reflection at the liquid–air interface confines the excitation beam and the scattered light. An Al mirror at the end of the laminar flow reflects the Raman signal to escape through the pinhole (pink) for collection at the microscope objective. Hence, the total reflection improved the efficiency of the generation and collection of Raman signals. The Al mirror was also used to fix the length of the laminar flow to 15 mm.

**Figure 1:**
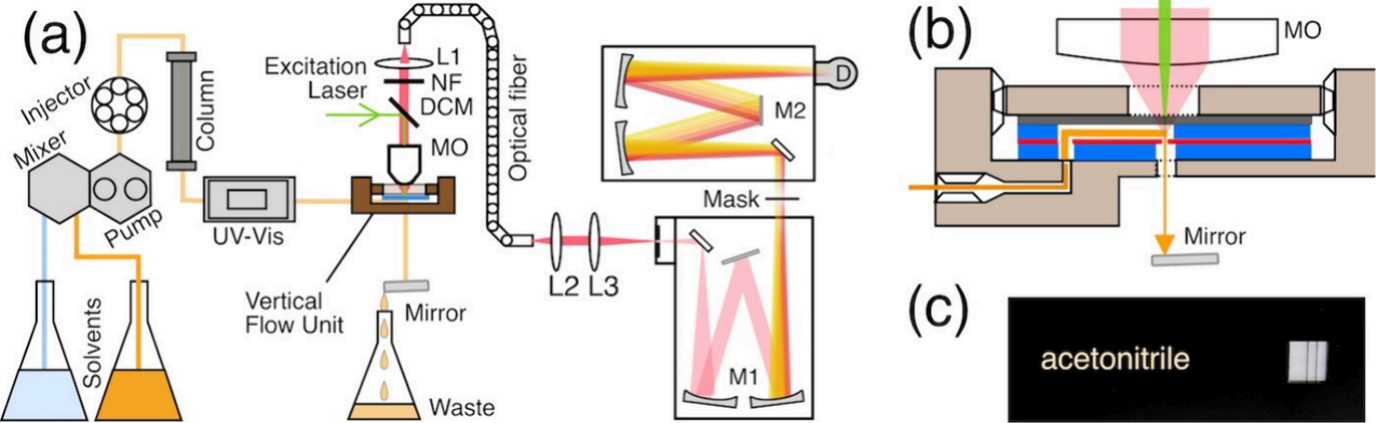
(a) Diagram of the HILIC-Raman spectrometer. DCM, dichroic mirror; L1–3, lenses; M1, monochromator 1; M2, monochromator 2; MO, microscope objective; NF, notch filter. (b) Diagram of the VF unit for organic solvents, consisting of a holder (brown), a coverslip (black), silicone sheets (blue), and a plastic film (red). Orange: flow route of the sample liquid, green: incident beam; red: Raman scattered light. (c) Photograph of the spatial line rejection mask designed to eliminate the Raman lines of ACN.

Through a notch filter (NF01-532U-25, Semrock) to reject the Rayleigh line, the Raman scattered light is delivered to the monochromator via a round-to-linear optical fiber (BFL105HS02, Thorlabs). This fiber was selected to convert the round image of the pinhole on the VF unit into a quasi-rectangular form and maximize the throughput at the entrance slit of the spectrometer.

The Raman spectrometer is designed in the laboratory. It consists of a 532 nm laser (CNI, China), two monochromators (M1, Shamrock SR-303i, Andor (F/4); M2, ISA HR320, Horiba-Jobin Yvon (F/4.4)), and an EMCCD detector (ProEM, Teledyne Princeton) attached to the exit port of M2. The two monochromators are positioned in tandem. Whereas the entrance slit of M1 is set to 20 μm, the other slits are open. M1 is used to monochromate the Raman signal. The grating of M2 is set to reflect the zeroth-order diffracted light (i.e., used as a mirror). The incident light from the entrance slit is transferred to the CCD detector at the exit slit.

A spatial line rejection mask is placed at the focal plane between M1 and M2. This mask was prepared by laser machining a 0.3 mm-thick plastic plate. A barcode pattern was designed to shade the solvent bands by considering the Raman spectral pattern and the CCD dimensions (13.3 mm × 13.3 mm) (Figure 1c).

### Conditions

For the HILIC-Raman experiment, an aqueous solution of 65% (v/v) acetonitrile (ACN) was selected as the running solvent. ACN is suitable for the HILIC-Raman measurement because its Raman band is sharp, and its overlap with the Raman bands of solutes is small, i.e., the spectral window is broad. Chromatography was performed under isocratic conditions. The flow rate was 3 mL min^–1^. Raman spectra were recorded every 0.4 s for 55 min after sample injection (*t* = 0 min). Ten spectra were averaged to obtain each spectrum in the data matrix. The data collected at 30–55 min were analyzed. The sample concentrations were set individually in each experiment (see below). The injected volume was 1 mL.

### Samples

Sugars were purchased [d-fructose (TCI, Japan), d-glucose and d-sucrose (Sigma-Aldrich, MO), d-maltose and d-trehalose (Fisher Chemical, MA)] and used without further purification. Honey was purchased from a local bee farmer in Hsinchu, Taiwan. One g of honey was dissolved in 20 mL of solvent and centrifuged at 16100 rcf for 15 min before the measurement.

### Data Analysis

#### Singular Value Decomposition (SVD)

Two-dimensional (2D) Raman data, plotted along temporal and spectral axes, were obtained as an *m* × *n* matrix (M) and then decomposed into a product of three matrices by applying SVD analysis.

1 $$M=U^{◦}{\mathrm{WV}}^{◦T}$$

where U° (*m* × *n* matrix) includes the spectral components, W (*n* × *n* diagonal matrix) the singular values (SVs), and V° (*n* × *n* matrix) the temporal components. The *i*-th SV (given as the *ii*-th component of W, *w*_*ii*_) represents the contribution of the *i*-th vectors in U° and V°. By analyzing a plot of *w*_*ii*_, we determined the number of significant components predominant among the temporal and spectral components in the Raman data.

#### Reconstruction

When the *m*_1_ components were predominant, the principal *m*_1_ vectors in U° and V° and the corresponding *m*_1_ SVs in W (U°_*m*1_, V°_*m*1_, and W_*m*1_, respectively) in eq 1 were used to approximate M, and an *m*_1_ × *m*_1_ regular matrix K and its inverse matrix K^–1^ were used to convert U°_*m*1_ and V°_*m*1_ into U_*m*1_^′^ and V_*m*1_^′^, i.e.,

M≈U◦m1Wm1V◦m1T=(U◦m1K)(K−1Wm1V◦m1T)=Um1′Vm1′T 2

The elements of K were chosen iteratively to minimize the square of the difference between U’_*m*1_ and a matrix in which each vector represented the spectral pattern of model compounds.

## Results and Discussion

Figure 2 shows the Raman spectra of glucose in 80% ACN (and 20% water) solutions. All measurements were conducted using VF by flowing the sample solution (the HPLC setup was not used). We compared three measurement conditions: (a) with a capillary glass as the sampling cell, (b) with the VF sampling unit, and (c) with the VF sampling unit and the spatial mask. Notably, the VF unit with the 60-μm pinhole enhanced the Raman signal by up to 168-fold.^14^ The first row shows the data recorded for the 50 mM glucose solution (solid line) and the solvent (dashed line). The exposure time was the longest but was less than the saturation value, and the signal intensity was almost the maximum count possible for the detector (65,536). The two spectra agree with each other. The inset magnifies the data in the 950–1,250 cm^–1^ range. The difference at 1,130 cm^–1^ is attributed to the Raman band of glucose. The spectra in Figure 2c1 differ from those in Figure 2a1,b1 because the Raman bands of the solvent (marked) are eliminated by the spatial mask. This allowed a longer exposure time, and thus, the signal intensity in the inset was nearly four times greater.

**Figure 2:**
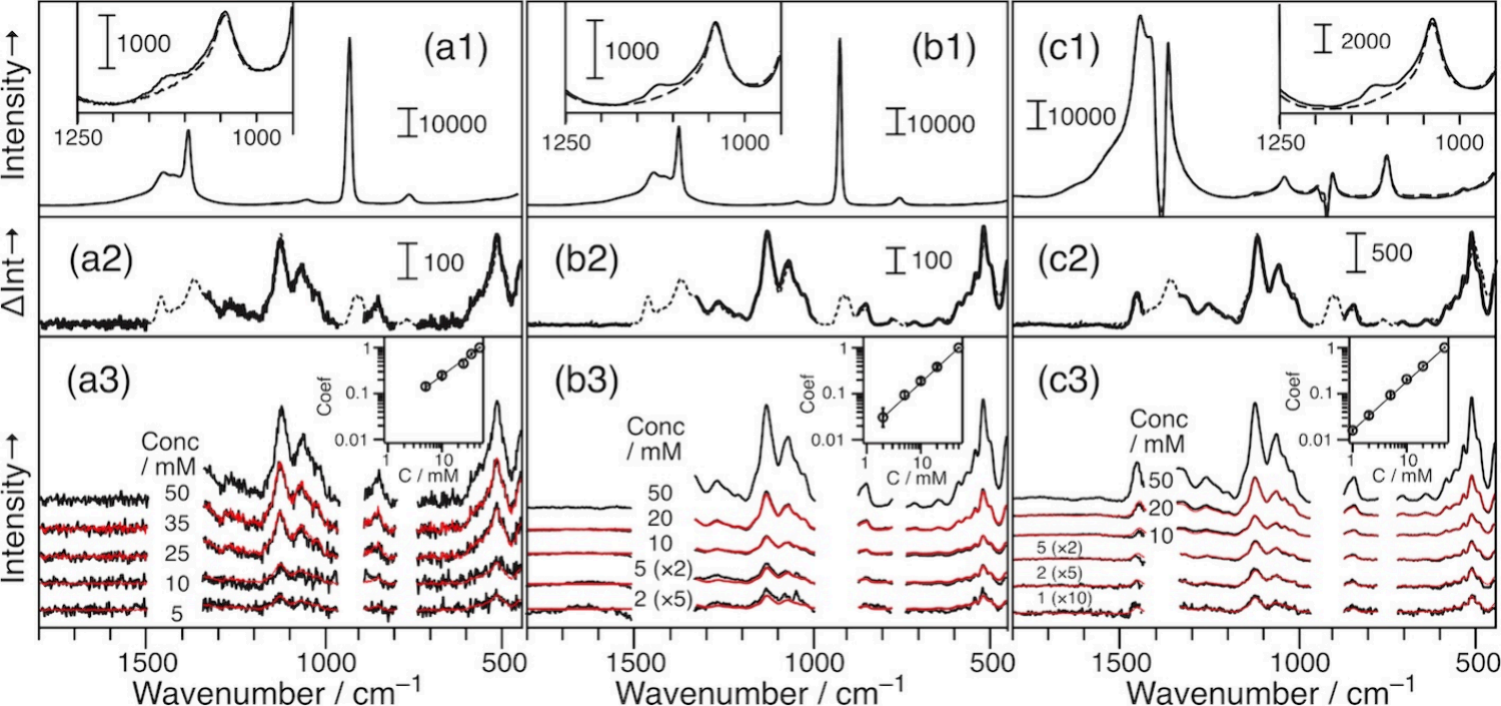
Raman spectra of glucose in 80% ACN measured with a capillary glass (a), via VF (b), and via VF with a spatial mask (c). The spectra of 50 mM glucose solution (solid line) and the solvent (dashed line) are compared in the first row, and their difference (solution spectrum minus solvent spectrum) is compared with the Raman spectrum of 50 mM glucose in an aqueous solution (second row; the intensity is matched up). The concentration dependence of the spectral pattern of glucose is shown in the third row (the concentration is shown therein; the red lines show the results of a fitting analysis). The inset shows the log–log plot of the relative intensity of Raman signals versus the concentration.

The second row depicts the difference between the two spectra, i.e., the solution spectrum minus the solvent spectrum. The peak height in Figure 2a2,b2 are 200–250, and it is ∼ 1,000 in Figure 2c2 because the exposure time increases. The difference spectra for the three cases agree with the Raman spectrum of the aqueous glucose solution subjected to VF (dashed line in the second row), demonstrating the successful Raman measurement of 50 mM glucose in 80% ACN. Some data points in the difference spectra were erased to remove the residual noise when the solvent bands were subtracted. The gap at approximately 1,400 cm^–1^ in Figure 2c2 is smaller than those in the other figures, suggesting that the spatial mask removes the solvent band effectively.

The third row shows the Raman spectrum of Glu. The concentration was 5–50 mM for the conventional measurements (Figure 2a3), 2–50 mM for the VF measurements (Figure 2b3), and 1–50 mM for the VF measurements with the spatial mask (Figure 2c3). The VF method improved the signal-to-noise (S/N) ratio of the spectra.

The relative intensity of the Raman bands at each concentration was evaluated by fitting the spectrum of 50 mM Glu. The multiplication coefficients used for this fitting analysis were obtained as the average values of triplicate results, and these values are plotted against the concentration in the insets of Figure 2a3,b3,c3. The results of a regression analysis explained the linear concentration dependence of the Raman signal intensity for all samples (*R* ^2^ > 0.98). From the parameters used in the linear regression analysis, the limits of detection (LOD) and quantification (LOQ) are determined to be 5.34 mM and 17.5 mM for the conventional measurements, 0.56 mM and 1.87 mM for the VF measurements, and 0.38 mM and 1.27 mM for the VF + SM measurements. Thus, the VF technique with the spatial mask improved the LOD and LOQ values by eliminating the large Raman bands of the solvent.

Next, we applied 1 mL of a solution of 200 mM Fru in 65% ACN and examined the elution time under isocratic conditions. Figure 3a shows the 2D HILIC-Raman matrix data 30–55 min after the injection. After subtracting the solvent bands, the matrix was decomposed into the product of three matrices via SVD analysis (see the Experimental Section for details).

**Figure 3:**
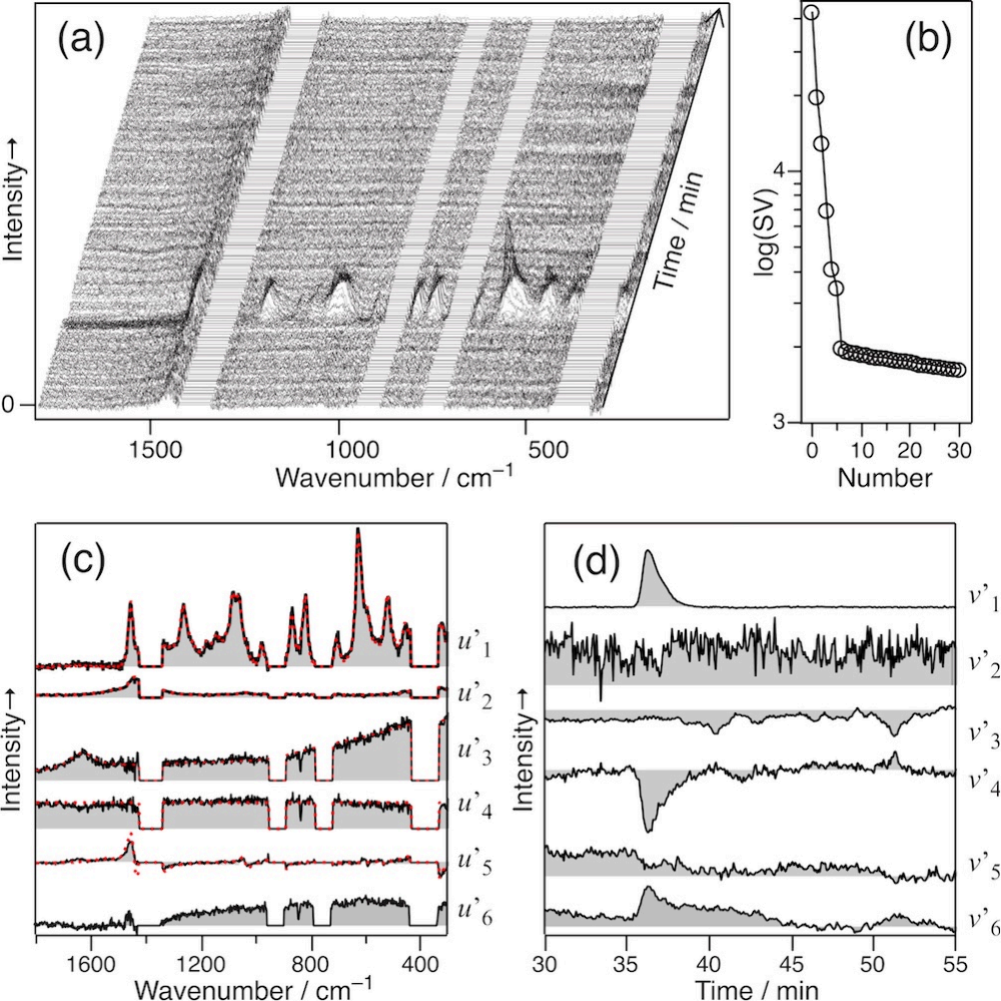
(a) 2D HILIC-Raman spectra of 200 mM fructose in an aqueous 65% ACN solution. (b) Plot of the SVs. The largest 30 values are shown. The six vectors of *u*′ (c) and *v*′ (d). The red lines in (c) show the model spectra of fructose (top), ACN, and H_2_O, as well as the baseline and the peak shift of ACN (fifth row).

The SV plot (Figure 3b) contains six significant components (*m*_1_ = 6 in eq 2). Accordingly, we generated U°_6_ and V°_6_, each consisting of six significant vectors: *u*_1_–*u*_6_ in U° and *v*_1_–*v*_6_ in V°. A linear combination of these vectors transformed U°_6_ and V°_6_ into U’_6_ and V’_6_ (eq 2). Figure 3c,d show the six derived vectors in U’_6_ and V’_6_, respectively. The six vectors *u*’_1_–*u*’_6_, obtained by selecting the elements of K (eq 2) by the least-squares fitting, effectively reproduce the expected Raman spectra of Fru, ACN, and H_2_O, as well as a constant background and the peak shifts of the Raman bands of ACN (Figure 3c, red).

Because *u*’_1_ represents the spectral pattern of Fru, *v*’_1_ corresponds to an elution curve. The elution time is found to be 36.4 min. *u*’_2_ and *u*’_3_ reproduce the model spectra of ACN and H_2_O, respectively. For clarity, the intensities of these model spectra are multiplied by 0.025 and 0.02, respectively. Consequently, *v*’_2_ and *v*’_3_ are magnified by factors of 40 and 50, respectively. *v*’_2_ and *v*’_3_ fluctuate around the zero line; the temporal instability of the direction of VF causes the instability of the solvent band intensity. The baseline is lowered when Fru is eluted (Figure 3a), as also shown by *v*’_4_ in Figure 3d. This observation could be attributed to the temporal increase in the refractive index of the eluate and the resultant increase in the divergence angle, which decreased the signal intensity detected by the microscope objective with a fixed NA, and/or a decrease in the solvent fraction upon the elution of Fru. Additionally, the baseline lowering was observed because most of the solvent band was subtracted in the 2D data, and a slight change in the intensity remained visible. Finally, the sixth vector is obtained as the residual. This component seemed to correspond to a baseline drift.

The same analysis was carried out for solutions of Glu (160 mM), Suc (100 mM), Mal (100 mM), and Tre (100 mM) in 65% ACN. The elution peak was detected at 39.3 min for Glu, 42.7 min for Suc, 46.4 min for Mal, and 47.9 min for Tre (Table 1; Figure S1).

**Table 1:** Elution Time and LOD and LOQ Values of Each Sugar in HILIC-Raman Measurements

	Elution/min	LOD/μg mL^–1^ (/mM)	LOQ/μg mL^–1^ (/mM)
Fru	36.4	61 ± 11 (0.34 ± 0.06)	204 ± 38 (1.1 ± 0.2)
Glu	39.3	68 ± 31 (0.38 ± 0.17)	226 ± 103 (1.3 ± 0.6)
Suc	42.7	74 ± 17 (0.22 ± 0.05)	245 ± 58 (0.7 ± 0.2)
Mal	46.4	68 ± 12 (0.20 ± 0.04)	227 ± 41 (0.7 ± 0.1)
Tre	47.9	81 ± 43 (0.24 ± 0.13)	269 ± 143 (0.8 ± 0.4)

The observed elution peak was broad, and tailing occurred because a substantial amount of sample was used. An unexpected disruption of the sample solution in the VF unit is not likely. The volume of the laminar flow was 0.17 mm^3^, as the liquid core waveguide (LCW) was 60 μm in diameter and 15 mm in length. The sample flowing at 3 mL min^–1^ passed through this volume in 3.4 ms, and the sample did not stay in the LCW to cause tailing.

We analyzed a mixed solution of the five sugars (25 mM for each) using HILIC-Raman and examined the resolving power. Figure 4 shows the 2D HILIC-Raman result. The spatial mask eliminates the Raman bands of ACN, and data for the masked region are lacking (Figure 4a). Figure 4b shows a plot of the 30 largest SVs. We employed nine components by considering the significance of each SV. Then, U°_9_ and V°_9_, containing the nine vectors, are transformed to U’_9_ and V’_9_ by selecting the elements of K (eq 2) to minimize the difference between the vectors in U’_9_ and the expected spectral patterns of the five sugars, the solvents, the peak shift of the Raman bands of ACN, and the baseline. The Raman spectra of the five sugars were separately recorded as the model spectra.

**Figure 4:**
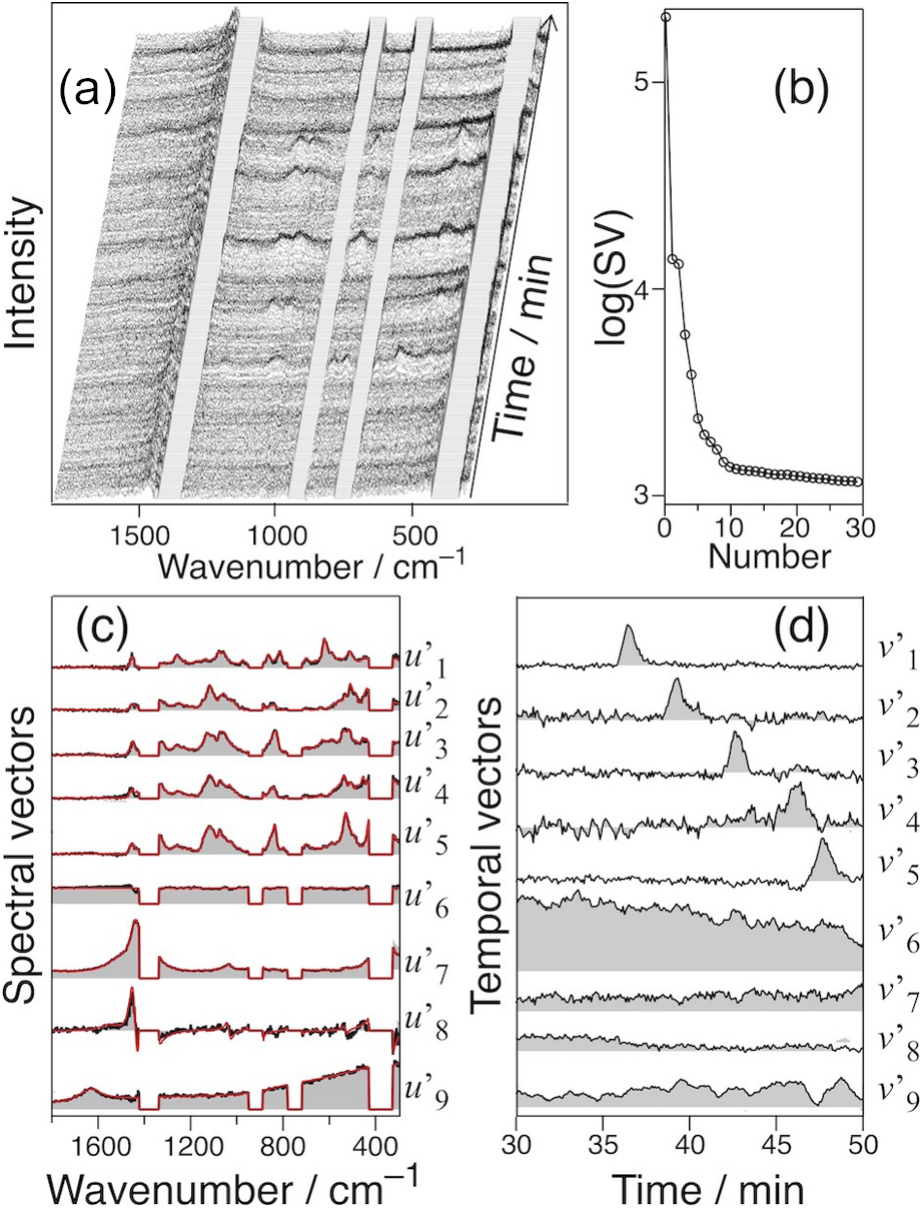
(a) 2D HILIC-Raman spectra of the mixture of five sugars in a 65% ACN aqueous solution. (b) Plot of SVs. The highest 30 points are shown. Nine reconstructed vectors of *u*′ (c) and *v*′ (d) are presented; the red lines in (c) show the model spectra of fructose (top), glucose, sucrose, maltose, trehalose, and ACN, as well as the peak shifts of ACN and H_2_O (bottom).

The vectors *u*’_1_–*u*’_5_ and *v*’_1_–*v*’_5_ represent the spectral and temporal patterns, respectively, of Fru, Glu, Suc, Mal, and Tre. *u*’_1_–*u*’_5_ excellently reproduces the Raman spectra of these sugars (red traces in Figure 4c). Accordingly, *v*’_1_–*v*’_5_ represents the elution curves of these components. The sugars elute over 11.3 min (from Fru at 36.4 min to Tre at 47.7 min) without severe overlap. This resolving power exceeds RPLC-Raman, where the five sugars eluted over a range of 1.8 min (Glu at 17.6 min and Suc at 19.4 min).^16^ This higher resolving power demonstrated the advantage of HILIC as expected. Raman detection allowed the unambiguous identification of the eluted sugars. The elution times did not differ from those in an independent analysis of each sugar (Table 1). We confirmed the reproducibility of the elution times of the sugars in the HILIC-Raman analysis.

The HILIC-Raman signal intensity was examined for Suc at different concentrations. Figure 5a shows the matrix data combining five sets of data over the range of 30–55 min at 25, 20, 15, 10, and 5 mM (the data sets correspond to regions I–V along the time axis). Each data set includes the elution peak of Suc at 42.7 min as shown in Figure 4d. The SV plot was obtained (Figure 5b), and the reconstruction was executed with the eight significant components. By transforming the matrices (eq 2), we obtain the vectors in U’_8_ and V’_8_ (Figure 5c,d). The coefficients for the transformation are selected iteratively such that the vectors in U’_8_ reproduced the Raman spectral pattern of Suc (*u*’_1_), ACN (*u*’_2_), the first derivative of ACN (*u*’_3_), the constant component (*u*’_4_), and H_2_O (*u*’_5_). *u*’_6–8_ are attributed to changes in the baseline. *v*’_1–8_ represents the corresponding temporal behaviors in Figure 5d. The amplitude of *v*’_*n*_ is set to unity (accordingly, that of *u*’_n_ is changed: *u*’_2_, *u*’_4_, *u*’_6_, *u*’_8_ are magnified 0.3, 8, 0.4, and 0.5 times, respectively). The magnitude of *u*’_*n*_ indicates the contribution of each component in the data matrix.

**Figure 5:**
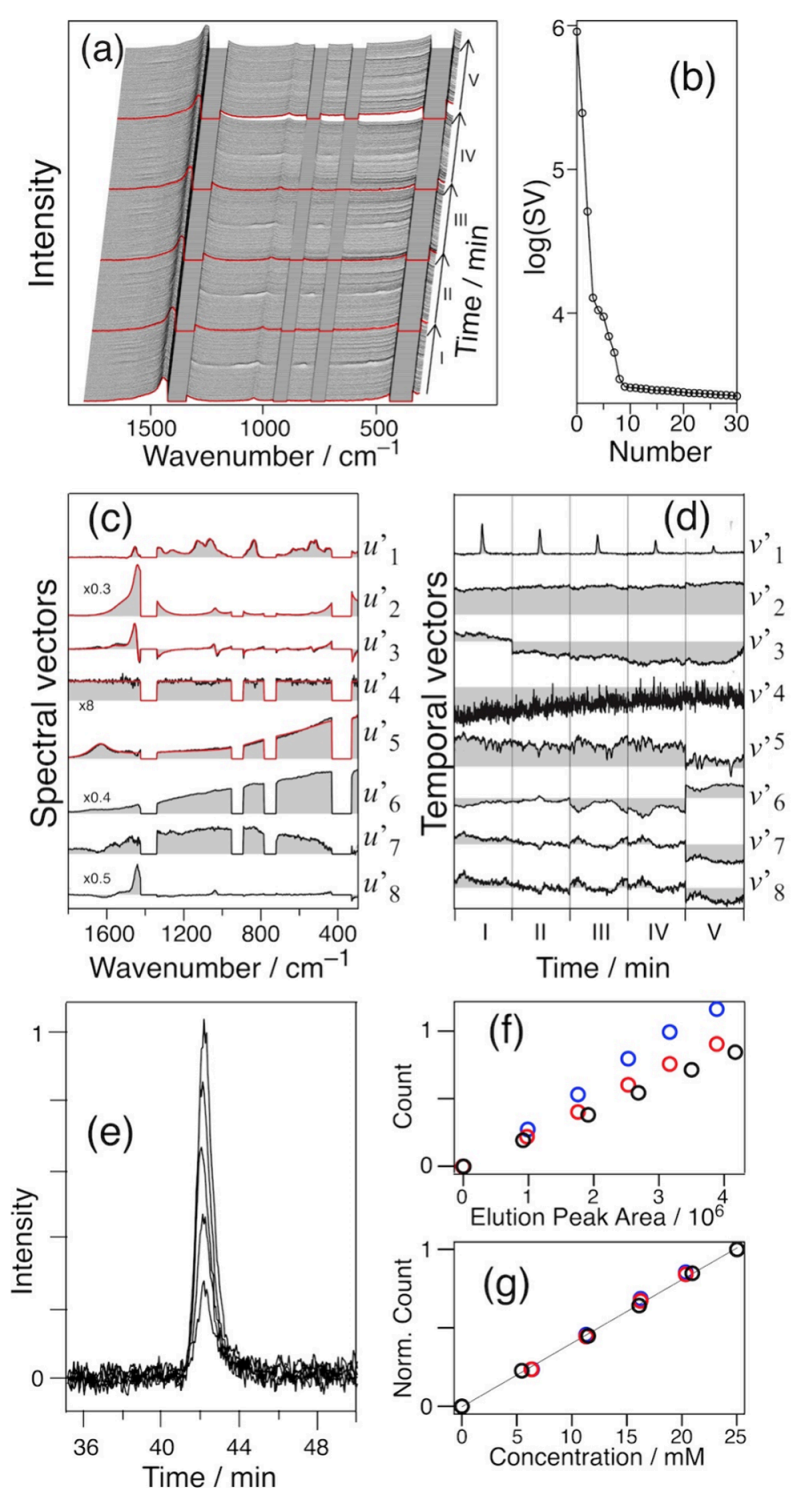
(a) Combined 2D HILIC-Raman spectra of sucrose at 5–25 mg mL^–1^. Red trace indicates the first spectrum of each set. (b) Singular value plot. Nine reconstructed vectors of *u*′ (c) and *v′* (d) are presented; the red lines in (c) show the model spectra of sucrose (top), ACN, the peak shift of ACN, a constant component, H_2_O, and three baseline components (bottom). *u*′_2_, *u*′_4_, *u*′_6_, and *u*′_8_ are magnified 0.3, 8, 0.4, and 0.5 times, respectively, for clarity. (e) Superposition of the elution peaks in *v*′_1_. The Raman intensity is plotted against the elution peak area of the chromatogram (f). (g) is the plot normalized by the values at 25 mM. Three independent data sets are shown with different colors in (f, g).

*v*’_1_ exhibits five peaks of the five data sets (Figure 5d). The peaks in regions I–V are plotted against the elution time (Figure 5e). The peak intensity decreases in proportion to the concentration. Figure 5f is a plot of the Raman peak intensity, obtained by the fitting analysis with a Gaussian function, against the elution peak area of the chromatogram at 210 nm. The results of the three independent experiments are shown herein. Among the plots of the three data sets, the deviation along the *x*-axis is due to errors in the applied sugar amount. The deviation along the *y*-axis originates mainly from the fluctuation of the signal enhancement factor of the VF method. This factor is due to the quality of the pinhole and the resultant quality of the laminar flow surface where the total internal reflection occurs. Note that the signal enhancement factor is identical in each data set because the same pinhole is used. The *R* ^2^ values of the linear regression analysis on the three data sets are 0.997, 0.997, and 0.999, respectively, showing excellent linearity.

The values in the x and y axes are normalized by those at 25 mM, respectively (Figure 5g). The normalized data plots coincide well and are reproducible. These data sets fit well with the linear function:

$$\left[\mathrm{Norm.}\;\mathrm{Raman count}\right]=\left(-0.005\pm 0.007\right)+\left(1.02\pm 0.01\right)\times \left[\mathrm{Norm.}\;\mathrm{Area}\right],\left(R^{2}=0.999\right)$$

Considering the error of each parameter, the normalized peak area is derived from the normalized Raman count within an error of 1.2%, showing the accuracy of this analysis.

From the coefficient and the error of the linear regression analysis, the LOD and LOQ of Suc (molecular weight (MW) = 342.3) were determined to be 0.22 ± 0.05 mM (74 ± 17 μg mL^–1^) and 0.7 ± 0.2 mM (245 ± 58 μg mL^–1^), respectively. Table 1 and Figure S2 present the results for each sugar. Despite the limited solubility of sugars in the ACN-water mixture, the LOD value improved 30–100 times relative to those reported in our previous RPLC-Raman study with the C_18_ column, a compact Raman spectrometer, and the VF method^16^ [where the LODs were 6.3 ± 0.2 mg mL^–1^ for Fru, 2.8 ± 0.1 mg mL^–1^ for Glu, 9.0 ± 0.2 mg mL^–1^ for Suc, 6.3 ± 0.1 mg mL^–1^ for Mal, and 2.9 ± 0.1 mg mL^–1^ for Tre].

The improvements of the Raman spectrometer and the VF method raised the sensitivity. A possible reason was the use of shorter-wavelength excitation (785 nm in RPLC-Raman vs 532 nm in HILIC-Raman), as the number of scattered photons in the Raman process depends on λ^–3^. Besides, the replacement of the CCD detector improved the quantum efficiency (60–80% at 800–940 nm vs 90% at 540–600 nm) an decreased the noise level owing to less dark current (50 e-/pixel/s vs 0.04 e-/pixel/s), thermal noise (15 °C vs – 55 °C), and read-out noise from the detector (6 e^–^ vs <1 e^–^). The improved performance is also due to the improved signal collection efficiency due to an optimization of the NA value of the microscope objective (0.64 vs 0.80) and the optimization of the pinhole size in the VF unit, which maximized the signal enhancement factor (90-fold at 100 μm^13^ vs 168-fold at 60 μm^14^). The spectral pattern became more distinguishable because HILIC set the elution peaks apart from each other, and the spatial mask eliminated the unnecessary Raman bands.

It is intriguing to apply the HILIC-Raman technique to a natural product. We analyzed the sugar content in honey. Figure 6a shows the obtained 2D data, where the spatial mask shuts off the Raman bands of ACN. Even though the SV plot indicates the predominance of eight components (Figure 6b), we fed 11 vectors for further analysis to find minor sugar component(s). Careful reconstruction allowed us to explain the 2D data by considering the 11 spectral (*u*’_1–11_ in Figure 6c) and temporal vectors (*v*’_1–11_ in Figure 6d). The vectors *u*’_1_ – *u*’_5_ correspond to the Raman spectra of Fru, Glu, Suc, Mal, and Tre, respectively. The others are the background signals. The elution peaks of Fru, Glu, and Suc appear at 36.3, 39.0, and 42.1 min, respectively, consistent with the other results (Table 1).

**Figure 6:**
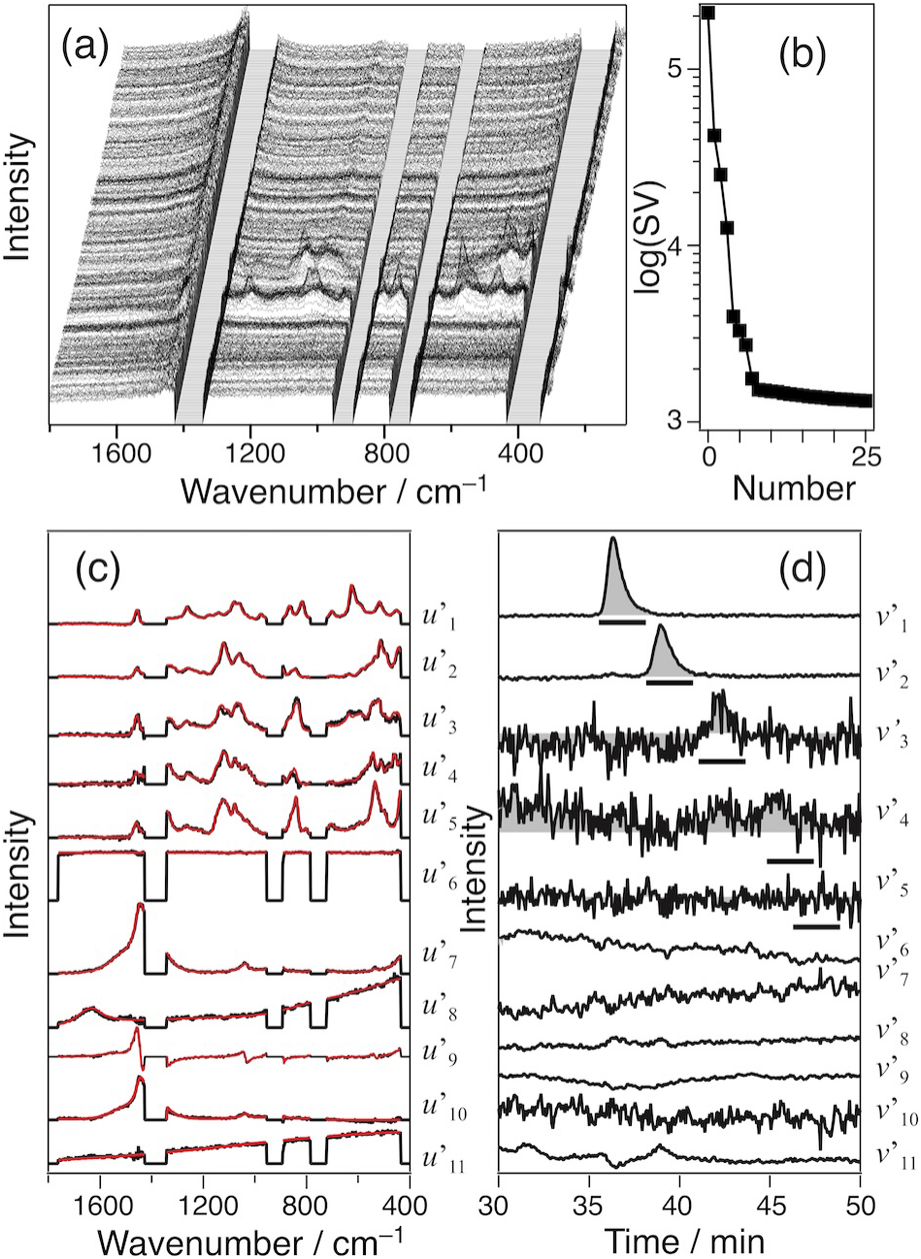
Results of HILIC-Raman sugar analysis in honey. 2D data (a), singular value plot (b), and the spectral (c) and temporal (d) vectors obtained from the reconstruction. *v*′_3–5_ are magnified ten times. Bars in (d) indicate the elution time of each sugar.

The sugars in honey were quantified by comparing the elution peak areas of *v*’_1–3_ and those obtained from 1 mL of 200 mM Fru, 160 mM glucose, and 100 mM Suc (Table S1). The peak area was estimated by integrating the underlined period of the elution curve in Figure 6d [35.3 – 38.0 min for Fru, 38.0 – 40.7 min for Glu, 40.7 – 43.3 min for Suc, 44.7 – 47.3 min for Mal, and 46.0 – 49.0 min for Tre]. From the triplicate experiments, the weight% of each sugar in honey is determined as Fru 39.9 ± 0.6, Glu 26.5 ± 1.8, and Suc 0.9 ± 0.5 (Table S1). Mal and Tre are not detected within the error, suggesting their concentration is less than the LOD. The sugar content was also examined using LC with the refractive index detector. The obtained concentration (wt %) was 68.8 ± 0.5 for Fru + Glu and 1.4 ± 0.5 for Suc (Table S1). Our result (Fru + Glu 66.4 ± 1.9, Suc 0.9 ± 0.5) agreed with these values within the errors. Hence, our method offers a novel approach for the quantitative analysis of the solutes in the LC eluate. The determined sugar content was consistent with other reports, where the contents of Fru, Glu, and Suc are 30 – 45 wt %, 21 – 37, and 0.1 – 8, respectively (Mal and Tre are not detected in most cases).^23^

The quantitative analysis of glucose is of particular interest owing to its relevance to diabetes (the homeostatic concentration of blood glucose is 4–7 mM^24^). When Raman spectroscopy was used with machine learning techniques, glucose concentrations were determined with a root mean squared error of prediction in the sub-mM range.^25^ Our results are comparable to these previous reports (Table 1). Notably, this comparable performance was achieved with the HILIC-Raman data that contains the glucose spectra only in a short elution time, possibly because of the enhanced signal intensity with the VF technique. Analysis of the Raman data enhanced by the VF with machine learning procedures is expected to improve the ability to detect sugars at lower concentrations.

To date, various methods have been adopted to detect sugars in LC eluates, e.g., refractive index detection (RID), with which LOD of Glu of 2.67 μg/mL^26^ or 2.1 μg/mL^27^ has been achieved; pulsed amperometric detection of anionic sugars in the eluate of anion exchange chromatography;^28^ detection of light scattering (via an evaporative light scattering detector, ELSD), with which LOQ of Glu of 0.08 g/100 g^29^ (i.e., 80 μg/mL) has been achieved; and charged aerosol detection (CAD)^30^ of solute particles generated by nebulization and evaporation of the solvent. In these detection techniques, physical quantities are measured as scalar values and different compounds are not distinguishable. The reliability of the quantitative analysis may be lowered by the interference of other compounds.

Spectral detection methods provide authentic information to identify eluted molecules owing to the uniqueness of the spectral pattern of each compound. Absorption in the vacuum-UV region, typically below 200 nm, can be used to distinguish Fru, Glu, and Suc under N_2_ purge conditions.^31^ The LOD value at 190 nm has been reported to be 6.3 μg/mL.^27^ A drawback of the Raman detection is in the sensitivity, as the LOD value observed in the present experiment (68 μg/mL; Table 1) was nearly 30 times greater than those reported for previous resonance ionization detection (RID) experiments (2.67 μg/mL^26^ or 2.1 μg/mL^27^). Hence, Raman spectroscopy is still not comparable in sensitivity to the established detection techniques described above. Another advantage of the Raman detection is its potential to access the dynamic nature of the conformation of sugar chains in solution as well as the static features such as the epimers, anomers, and the variety of the covalent linkage. The HILIC-Raman technique will be beneficial in revealing the complex nature of the sugar moieties in aqueous solution, e.g., those of biological samples, in the future. The LC-Raman and HILIC-Raman techniques could be applied more broadly if the Raman signal intensity is enhanced with the VF method more efficiently.

Finally, the green character and practicality were assessed. The AGREE score^32^ was 0.55, indicating relatively low greenness, and the BAGI score^33^ was 72.5 and judged to be ″practical″ (Figure S3). Low throughput (1 sample/hour) lowered these scores. The throughput is low because the VF method needs to use the large column (20 mm × 150 mm) to ensure the fast liquid flow at 3 mL min^–1^. These tests are valuable in learning the required properties of the experimental methods in analytical chemistry. The HILIC-Raman experiment will be improved to attain more greenness and practicality in the future.

## Conclusion

The HILIC-Raman technique was developed. This experiment involved the VF technique and the spatial line rejection mask that effectively eliminated the significant Raman signals attributed to organic solvents. We separated five sugars using HILIC-Raman. Compared with RPLC-Raman, the distribution of the elution time of five sugars was extended from 1.6 to 11.3 min, showing that HILIC was more suitable than RPLC for the sugar analysis. The LOD and LOQ values (Table 1) obtained for HILIC-Raman were 30–100 times better than those obtained for RPLC-Raman owing to the improvement of the Raman spectrometer and the VF method. HILIC-Raman measurement was applied to analyze the sugar content of honey. The weight% of Fru, Glu, and Suc was determined to be 39.9 ± 0.6, 26.5 ± 1.8, and 0.9 ± 0.5, respectively. Mal and Tre were not detected, suggesting their concentration was lower than the detection limit of the HILIC-Raman experiment. These results showed the successful performance of the HILIC-Raman experiment. By improving the sensitivity of Raman detection, this technique will be utilized extensively to separate and identify the components in mixtures such as natural products.

## Supplementary Material



## Notes

2D data of HILIC-Raman measurement of sugars and data analysis results; concentration dependence of the Raman signal intensity of sugars in HILIC-Raman results; results of sugar analysis in honey; assessment of the green characters and practicality (PDF)

## References

[1] Tanaka N., McCalley D. V. (2016). Core-shell, ultrasmall
particles, monoliths, and other
support materials in high-performance liquid chromatography. Anal. Chem..

[2] Uliyanchenko E. (2017). Applications
of hyphenated liquid chromatography techniques for polymer analysis. Chromatographia.

[3] Kuligowski J., Quintas G., Garrigues S., Lendl B., de la Guardia M., Lendl B. (2010). Recent advances in
on-line liquid chromatography - infrared spectrometry
(LC-IR). Trends Anal. Chem..

[4] Zong C., Xu M., Xu L.-J., Wei T., Ma X., Zheng X.-S., Hu R., Ren B. (2018). Surface-enhanced
Raman spectroscopy for bioanalysis:
reliablity and challenges. Chem. Rev..

[5] Zaffino C., Bedini G. D., Mazzola G., Guglielmi V., Bruni S. (2016). Online coupling of high-performance
liquid chromatography with surface-enhanced
Raman spectroscopy for the identification of historical dyes. J. Raman Spectrosc..

[6] Dallas T., Dasgupta P. K. (2004). Light at the end of the tunnel: recent analytial applications
of liquid-core waveguides. Trends Anal. Chem..

[7] Altkorn R., Koev I., Pelletier M. J. (1999). Raman performance
characteristics
of Teflon-AF 2400 liquid-core optical-fiber sample cells. Applied spectroscopy.

[8] Dijkstra R. J., Bader A. N., Hoornweg G. P., Brinkman U. A. T., Gooijer C. (1999). On-line coupling
of column liquid chromatography and Raman spectroscopy using a liquid
core waveguide. Anal. Chem..

[9] Thissen J., Klassen M. D., Constantinidis P., Hacker M. C., Breitkreutz B., Teutenberg T., Fischer B. (2023). Online coupling of size exclusion
chromatography to capillary enhanced Raman spectroscopy for the analysis
of proteins and biopharmaceutical drug products. Anal. Chem..

[10] Marquardt B. J., Vahey P. G., Synovec R. E., Burgess L. W. (1999). A Raman waveguide
detector for liquid chromatography. Anal. Chem..

[11] Zhou J., Chu W., Lu D., Liu J., Mao X., Na X., Zhang S., Qian Y. (2020). A novel 3D
printed negative pressure
small sampling system for bubble-free liquid core waveguide enhanced
Raman spectroscopy. Talanta.

[12] Hiramatsu H., Saito T. (2014). Vertical Flow Apparatus for Enhancement
and Efficient Collection
of Raman Signal. J. Raman Spectrosc..

[13] Li S.-C., Hiramatsu H. (2019). A vertical
flow method for sensitive Raman protein
measurement in aqueous solutions. Anal. Chem..

[14] Chen T.-H., Chen Y.-S., Hiramatsu H. (2024). Raman spectrometer
with vertical
flow method for solutions containing organic solvents. Spectrochim. Acta A: Mol. Biomol. Spectrosc..

[15] Lo Y.-H., Hiramatsu H. (2020). Online liquid
chromatography-Raman spectroscopy using
the vertical flow method. Anal. Chem..

[16] Weng L.-H., Hiramatsu H. (2023). Determination
of sugar content in honey using LC-Raman
and programmable pump-Raman methods. Analytical
Methods.

[17] Galant A.
L., Kaufman R. C., Wilson J. D. (2015). Glucose: detection and analysis. Food Chem..

[18] Tanaka T., Nakashima T., Ueda T., Tomii K., Kouno I. (2007). Facile discrimination
of aldose enantiomers by reversed-phase HPLC. Chem. Pharm. Bull..

[19] Rudd P. M., Gulle G. R., Kuster B., Harvey D. J., Opdenakker G., Dwek R. A. (1997). Oligosaccharide sequencing technology. Nature.

[20] Jandera P. (2011). Stationary
and mobile phases in hydrophilic interaction chromatography: a review. Anal. Chim. Acta.

[21] Armenta S., Lendl B. (2010). Capillary liquid chromatography with
off-line mid-IR and Raman micro-spectroscopic
detection: analysis of chlorinated pesticides at ppb levels. Anal. Bioanal. Chem..

[22] Bellissent-Funel M.-C., Hassanali A., Havenith M., Henchman R., Pohl P., Sterpone F., van der Spoel D., Xu Y., Garcia A. E. (2016). Water determines
the structure and dynamics of proteins. Chem.
Rev..

[23] White, J. W. ; Riethof, M. L. ; Subers, M. H. ; Kushnir, I. Composition of American honeys; US Dept. Agric. Technical Bulletin 1261, 1962; pp 1–124.

[24] Berger A. J., Itzkan I., Feld M. S. (1997). Feasibility
of measuring blood glucose
concentration by near-infrared Raman spectroscopy. Spectrochim. Acta, Part A.

[25] Wang Q., Pian F., Wang M., Song S., Li Z., Shan P., Ma Z. (2022). Quantitative
analysis of Raman spectra
for glucose concentration in human blood using Gramian angular field
and convolutional neural network. Spectrochim.
Acta Part A: Mol. Biomol. Spectrosc..

[26] Filip M., Vlassa M., Coman V., Halmagyi A. (2016). Simultaneous
determination
of glucose, fructose, sucrose and sorbitol in the leaf and fruit peel
of different apple cultivars by the HPLC-RI optimized method. Food Chem..

[27] Jalaludin I., Kim J. (2021). Comparison of ultraviolet
and refractive index detections in the
HPLC analysis of sugars. Food Chem..

[28] Eggleston G., Borges E. (2015). Multiple applications
of ion chromatography oligosaccharide
fingerprint profiles to solve a variety of sugar and sugar-biofuel
industry problems. J. Agric. Food Chem..

[29] Zhang Y., Zhang W., Hou J., He J., Li K., Li Y., Xu D. (2023). Determination of sugars
and sugar alcohols in infant
formula by high performance liquid chromatography with evaporative
light-scattering detector. J. Chromatogr. B.

[30] Hetrick E. M., Kramer T. T., Risley D. S. (2017). Evaluation
of a hydrophilic interaction
liquid chromatography design space for sugars and sugar alcohols. J. Chromatogr. A.

[31] Uchiho Y., Goto Y., Kamahori M., Aota T., Morisaki A., Hosen Y., Koda K. (2015). Far-ultraviolet absorbance
detection
of sugars and peptides by high-performance liquid chromatography. J. Chromatogr. A.

[32] Pena-Pereira F., Wojnowski W., Tobiszewski M. (2020). AGREE-Analytical
GREEnness metric
approach and software. Anal. Chem..

[33] Manousi N., Wojnowski W., Płotka-Wasylka J., Samanidou V. (2023). Blue applicability
grade index (BAGI) and software: a new tool for the evaluation of
method practicality. Green Chem..

