# Supplementary material for: Sugar Analysis Using Hydrophilic Liquid Chromatography Combined with Raman Spectroscopy
Source: Anal Chem. 2025 Jun 14;97(25):12981–8. doi: 10.1021/acs.analchem.4c06076 (PMC12224162; doi:10.1021/acs.analchem.4c06076)
Supplement: Supplementary file 1 [file ac4c06076_si_001.pdf]

Supporting Information

Sugar analysis using hydrophilic liquid chromatography combined with Raman spectroscopy

Yu-Sheng Chen and Hirotsugu Hiramatsu*

Department of Applied Chemistry and Institute of Molecular Science,
National Yang Ming Chiao Tung University, Hsinchu, 30010, Taiwan

Contents

1. Two-dimensional data of HILIC-Raman measurement of sugars and data analysis results
2. Concentration dependence of the Raman signal intensity of sugars in HILIC-Raman results
3. Results of sugar analysis in honey
   (1) Triplicate results of the sugar analysis in honey using HILIC-Raman
   (2) Result of sugar analysis in honey using LC with a refractive index detector (LC-RI)
4. Assessment of the green character and practicality

1. Two-dimensional data of HILIC-Raman measurement of sugars and data analysis results

Glucose, sucrose, trehalose, and maltose were separately analyzed via hydrophilic liquid chromatography combined with Raman spectroscopy (HILIC–Raman). Fig. S1 shows the results (A, glucose; B, sucrose; C, trehalose; D, maltose). The color image (top left) shows the 2D HILIC–Raman data plotted along spectral (horizontal) and temporal (vertical) axes. The Raman signal intensity is indicated by a rainbow color spectrum (purple–blue corresponds to high intensity, and red–orange corresponds to low intensity). The purple–blue features, corresponding to the high-intensity Raman signals, appears in a short time range in each panel (glucose at ~150 pixels, sucrose at ~200 pixels, trehalose at ~210 pixels, and maltose at ~200 pixels along the vertical axis), and these features represents the elution of the sugars in each experiment.

The top right panel shows the singular value (SV) plotted against the number of corresponding vectors. We find significant SVs of seven, six, six, and six in the cases of glucose (A), sucrose (B), trehalose (C), and maltose (D), respectively. The 2D data are approximated with the vectors having large SVs. The less significant vectors are rejected.

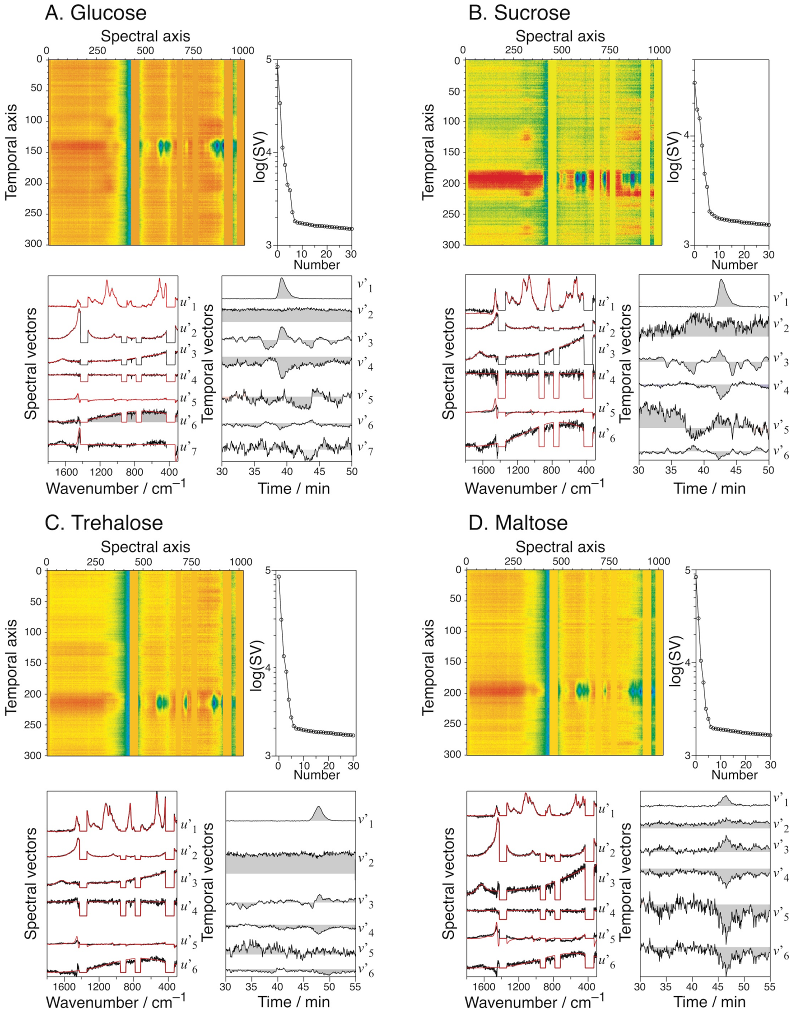

Figure S1. 2D HILIC–Raman data (left, top), singular value (SV) plot (right, top), and spectral (left, bottom) and temporal vectors (right, bottom) obtained from SVD and reconstruction analysis. The results for glucose (A), sucrose (B), trehalose (C), and maltose (D) are illustrated.

By transforming the matrices, we obtain sets of spectral (bottom left) and temporal vectors (bottom right). The spectral vectors are obtained by minimizing the difference between the experimental spectra and some model spectra. The model spectrum of each sugar is reproduced as u'_1 for glucose (A), sucrose (B), trehalose (C), and maltose (D), respectively.

Each panel represents the model spectra of acetonitrile (ACN), H_2O, and constant baseline as (u'_2, u'_3, u'_4). In addition, the baseline fluctuation is represented as u'_5 and the latter vectors.

The corresponding vector in V' represents the temporal behavior of each spectrum. We focus on the temporal behavior of the spectral components of each sugar (v'_1) for glucose (A), sucrose (B), trehalose (C), and maltose (D), respectively) because they correspond to the elution pattern of the sugars. From the analysis of the temporal components, we can determine the elution times to be 39.3 min for glucose, 42.7 min for sucrose, 47.9 min for trehalose, and 46.4 min for maltose.

2. Concentration dependence of the HILIC–Raman signal intensity of sugars

We prepared aqueous solutions of five sugars (fructose, glucose, sucrose, maltose, and trehalose) at different concentrations (25 mM, 20 mM, 15 mM, 10 mM, and 5 mM). The five obtained data matrices describing the concentration dependence of each sugar were combined to form one data matrix. This combined data matrix was analyzed using the SVD procedure and the reconstruction method.

Fig. S2 shows the reconstructed spectral (left) and temporal (right) vectors from triplicate experiments. During reconstruction, the model vectors (red traces in the left panels) of the sugars (at the position of u'_1), a constant component (u'_2), the Raman spectra of ACN (u'_3) and its first derivative as the peak shift (u'_4), H_2O (u'_5), and baselines (u'_6) are used. Depending on the number of significant SVs, some vectors are not used, and the optimum number and types of spectral components are employed in the analysis. The reconstruction is executed iteratively so that the temporal vectors reproduced the model vectors. The obtained results (black traces in the left panels) reproduce the model vectors successfully.

The corresponding spectral traces indicate temporal changes in the contribution of each spectral component. We focus on the temporal vectors of the sugar spectra (v'_1 in each panel). Because the data matrix consists of the five data sets, v'_1 exhibits five peaks corresponding to the elution of the five sugars used in these HILIC–Raman experiments.

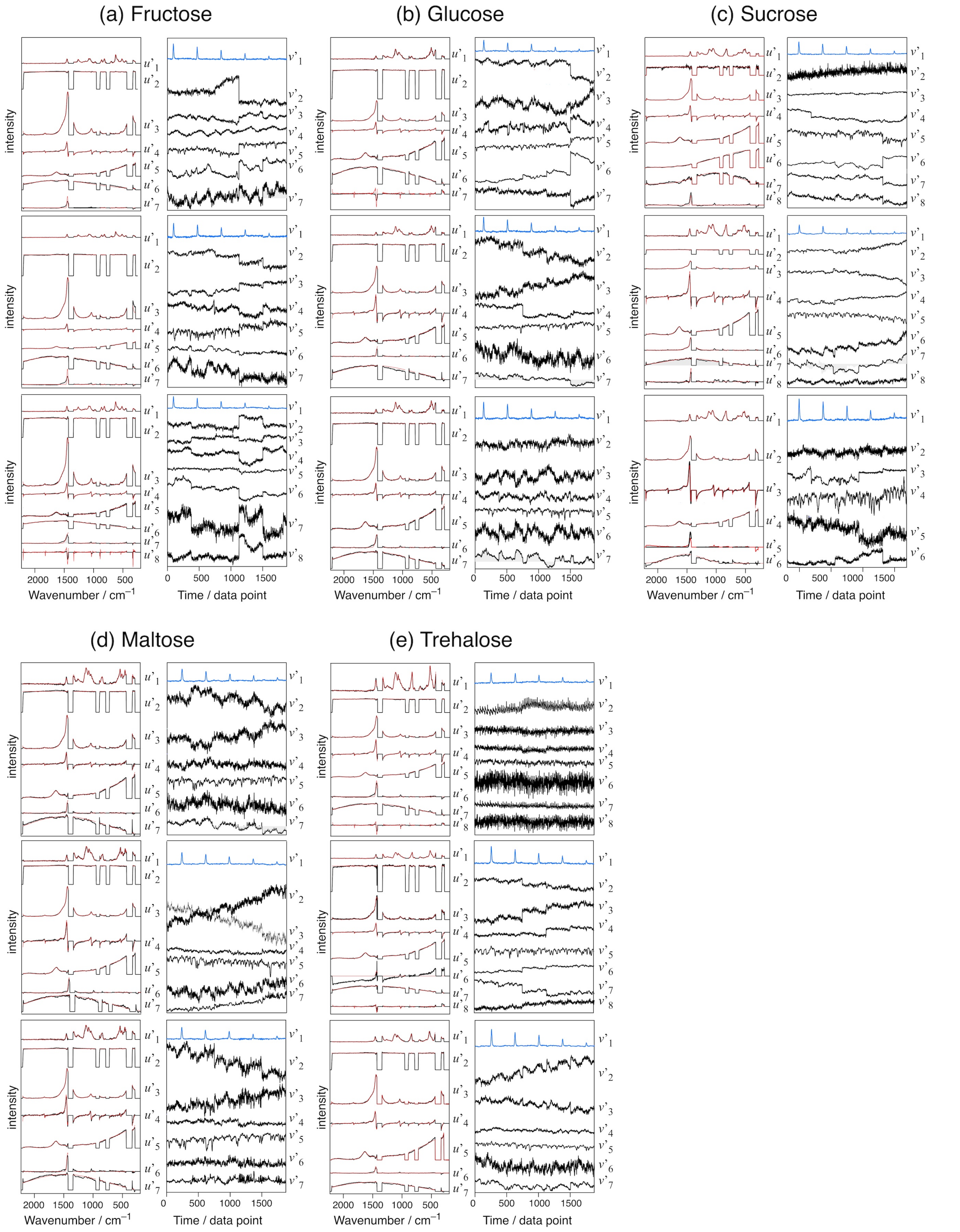

Figure S2. Results of the SVD reconstruction analysis of the combined data matrix containing results measured at five different concentrations: (a) fructose, (b) glucose, (c) sucrose, (d) maltose, and (e) trehalose.

3. Results of sugar analysis in honey

(1) Triplicate results of the sugar analysis in honey using HILIC-Raman

Figure S3 shows the results of the HILIC-Raman sugar analysis in honey. The reconstructed spectral (left) and temporal vectors (right) in the triplicate experiments are shown. The applied amount of honey is 56.2 mg, 53.3 mg, and 53.4 mg in 1 mL aqueous solution (samples 1, 2, and 3, respectively). Whereas the SVD analysis deduced the significance of eight vectors in each set, we adopted eleven vectors for the reconstruction to reproduce the minor sugar components, if any.

In the left panel, the vectors u'_{1-5} correspond to the Raman spectra of fructose, glucose, sucrose, maltose, and trehalose, respectively. The others ($u'_{6\text{-}11}$) are the baseline components. v'_{3-5} are magnified ten times to facilitate detecting the elution peaks.

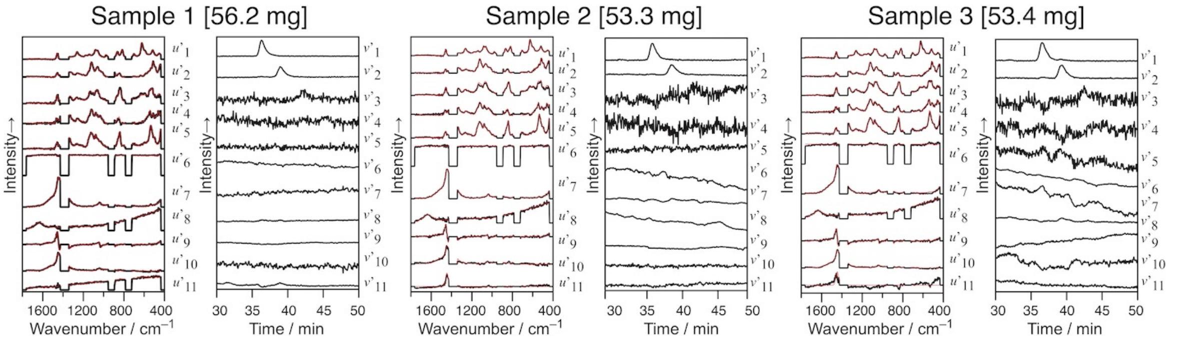

Figure S3. Results of HILIC-Raman sugar analysis in honey

The area of the elution peak is in proportion to the molar amount of the solute. The area of the elution peak was estimated by integrating the elution curve at 35.3 – 38.0 min for fructose, 38.0 – 40.7 min for glucose, 40.7 – 43.3 min for sucrose, 44.7 – 47.3 min for maltose, and 46.0 – 49.0 min for trehalose, where the elution peak appears (Fig. 4d).

The peak area was compared with those obtained from 1 mL of 200 mM fructose, 160 mM glucose, and 100 mM sucrose (Table S1 shows the weight and the integrated peak area), and the weight of each sugar in honey as well as their weight% in honey were calculated. By averaging the weight% of the three independent experiments, we deduced the average value of the weight% of each sugar in honey.

(2) Result of sugar analysis in honey using LC with a refractive index detector (LC-RI)

In addition to our HILIC-Raman method, the sugar content was analyzed separately by measuring the refractive index of the eluate of LC (LC-RI). The experiment was executed in SGS (Taipei, Taiwan) by following a standard procedure [CNS 1305-N5024, Taiwan]. Honey (5 g) was dissolved to 40 mL of H_2O and 25 mL of methanol, and then set to 100 mL solution by adding

H_2O. 10 µL of the honey solution was applied to LC after removing insoluble particles using a 0.45 µm filter. By the same procedure, three standard solutions containing fructose (2.000 g), glucose (1.500 g), and sucrose (0.25 g) were prepared separately. 80% acetonitrile aqueous solution was chosen as the mobile phase (1.3 ml min^{-1}). LC was performed using analytical hydrophilic column (4.6 × 250 mm) containing 5-7 µm amine-modified silica-gel. From a comparison of the area of the elution peaks of each sugar in honey with those of the standard solutions, the content of each sugar in honey was determined.

As shown in the report (attached below), the wt% of each sugar was determined as Fru and Glu 68.8 ± 0.5 and Suc 1.4 ± 0.5 (Table S1).

SGS

食品實驗室-台北
FOOD LAB-TAIPEI
測 試 報 告
Test Report

Test Item	Test Method	Results	LOQ/LOD (Note 3.)	Unit
Ⓚ Sucrose	CNS 1305 Honey (Date of Revision on 2023/07/05).	1.4	0.5	%
Ⓚ Glucose plus fructose content		68.8	0.5	%

3. If the testing item belongs to quantitative analysis then this column describes Limit of Quantification(LOQ); If the testing item belongs to qualitative analysis then this column describes Limit of Detection(LOD).

Table S1. Elution peak area and estimated amount and weight(wt) % of sugars in honey

		Standard [applied wt / mg]		Honey [applied wt / mg] sample 1 [56.2]	sample 2 [53.3]	sample 3 [53.4]	average wt%	LC-RI†
**Fru**	Area	56.5 [36.0]	Area	34.9	33.1	34.0		
			wt/mg	22.2	21.1	21.7		
			wt%	39.5	39.5	39.5	39.9 ± 0.6	[68.8 ± 0.5]‡
**Glu**	Area	53.1 [28.8]	Area	25.7	27.9	25.8		
			wt/mg	13.9	15.1	14.0		
			wt%	24.8	28.4	26.2	26.5 ± 1.8	[68.8 ± 0.5] ‡
**Suc**	Area	33.8 [34.2]	Area	0.665	0.197	0.641		
			wt/mg	0.67	0.20	0.64		
			wt%	1.2	0.4	1.2	0.9 ± 0.5	1.4 ± 0.5
**Mal**			Area	0.678	-0.684	-0.542		(not tested)
**Tre**			Area	0.048	0.182	-0.676		(not tested)

† tested at SGS [Taipei, Taiwan]. ‡ wt% of Fru plus Glu.

4. Assessment of the green character and practicality

In order to assess the green character and practicality of the developed method, we used AGREE [1] and BAGI [2] as the metric tools for this purpose.

Figure S3(a) shows the greenness score (AGREE). It was 0.55 and rather low, indicating that our method has only limited green characters. Among the features for the evaluation, the scores of No. 5, 7, 10, 11 are rather poor, showing the points to improve:

1. Sample treatment: on-line analysis
2. Sample size: sample amount 1 mL
3. In-situ measurement: on-line
4. Number of analytical processes: 3 or fewer
5. Automation & Minuarization: semi-automatic, not miniturized
6. Derivatization: no derivatization
7. Volume of analytical waste: 3mL/min x 60 min = 180 mL
8. Throughput: 6 samples determined in a single run (five sugars, acetonitrile), 1 sample/hour
9. Use of energy: LC
10. Source of reagents: some reagents are bio-based
11. Toxicity: yes, acetonitrile 100 mL
12. Safety of the operator: (not applicable)

Besides, Fig. S3(b) illustrates the score of BAGI, which is 72.5 and judged to be "practical." We chose the following options.

1. Type of analysis: Quantitative and confirmatory
2. Multi-or single-element analysis: Multi-element analysis for 2-5 compounds of the same chemical class
3. Analytical technique: Homemade instrumentation
4. Simultaneous sample preparation: 2-12
5. Sample preparation: Not required or on-site sample preparation if required
6. Samples per h: ≤1
7. Reagent and materials: Common commercially available reagents
8. Preconcentration: Preconcentration required. Required sensitivity is met with one-step preconcentration.
9. Degree of automation: Semi-automated with common devices (HPLC autosampler)
10. Amount of sample: <10mL food

Both scores are reduced with the low temporal throughput (≤ 1 sample / hour). At present, HILIC-Raman is time-consuming it requires a preparative column (20 mm in diameter, 15-20 cm in length) to make the flow rate high enough (3 mL / min) as to form a laminar flow in the vertical flow method. The use of an analytic column (e.g., 4.6 mm in diameter and 10 cm in length) could shorten the time for the single-run experiment. It would improve the efficiency of the experiments (No. 8 in AGREE and No. 6 in BAGI) and reduce the waste volume (No. 7 and 11 in AGREE), thereby improving the AGREE and BAGI scores. The experimental apparatus should be improved to achieve the better greenness and practicality.

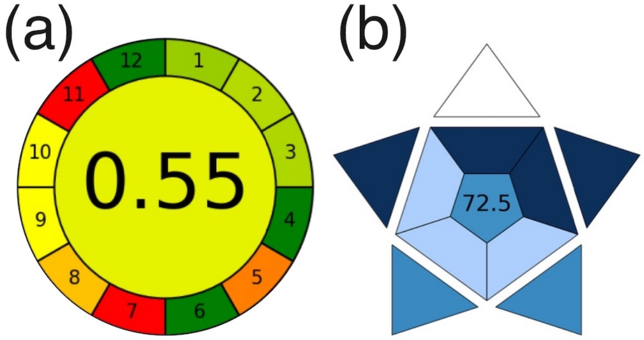

Figure S3. Scores of AGREE (a) and BAGI (b). See text.

References

[1] Pena-Pereira, F.; Wojnowski, W.; Tobiszewski, M. AGREE-Analytical GREEnness metric approach and software. *Anal. Chem.* **2020**, *92*, 10076-10082.

[2] Manousi, N.; Wojnowski, W.; Płotka-Wasylka, J.; Samanidou, V. Blue applicability grade index (BAGI) and software: a new tool for the evaluation of method practicality. *Green Chem.* **2023**, *25*, 7598-7604.
